# Non-Destructive Screening of Sodium Metabisulfite Residue on Shrimp by SERS with Copy Paper Loaded with AgNP

**DOI:** 10.3390/bios13060575

**Published:** 2023-05-25

**Authors:** Chao Yuan, Yanan Zhao, Xingjun Xi, Yisheng Chen

**Affiliations:** 1 State Key Laboratory of Biobased Material and Green Papermaking, Qilu University of Technology, Shandong Academy of Sciences, Jinan 250353, China; 2 College of Food Science and Engineering, Shanxi Agricultural University, Jinzhong 030801, China; 3 Sub-Institute of Agricultural Food Standardization, China National Institute of Standardization, Beijing 100191, China

**Keywords:** sodium metabisulfite, surface-enhanced Raman spectroscopy, copy paper, AgNPs, shrimp

## Abstract

In order to prompt the appearance of the shrimp color, sodium metabisulfite is frequently added in shrimp processing, which is, however, prohibited in China and many other countries. This study aimed to establish a surface-enhanced Raman spectroscopy (SERS) method for screening sodium metabisulfite residues on shrimp surfaces, in a non-destructive manner. The analysis was carried out using a portable Raman spectrometer jointly with copy paper loaded with silver nanoparticles as the substrate material. The SERS response of sodium metabisulfite gives two fingerprint peaks at 620 (strong) and 927 (medium) cm^−1^, respectively. This enabled unambiguous confirmation of the targeted chemical. The sensitivity of the SERS detection method was determined to be 0.1 mg/mL, which was equal to residual sodium metabisulfite on the shrimp surface at 0.31 mg/kg. The quantitative relationship between the 620 cm^−1^ peak intensities and the concentrations of sodium metabisulfite was established. The linear fitting equation was y = 2375x + 8714 with R^2^ = 0.985. Reaching an ideal balance in simplicity, sensitivity, and selectivity, this study demonstrates that the proposed method is ideally suitable for in-site and non-destructive screening of sodium metabisulfite residues in seafood.

## 1. Introduction

Sodium metabisulfite (sodium bisulfite), commonly known as “shrimp powder”, is often used as the blenching and preservative agent for shrimp processing. At the surface of shrimp, sodium metabisulfite generates reductive sulfite, resulting in significant color protection effects. More specifically, sulfite can effectively control the browning of shrimp color by inhibiting oxidase activity. Moreover, it can suppress the growth of microorganisms as well [1]. For these reasons, sodium metabisulfite is frequently added to shrimp in order to prompt the product’s appearance. However, this inevitably causes the problem of excessive residue. Remarkably, the toxicity of sodium metabisulfite to human beings had been experimentally evidenced, leading to strict restriction of it in seafood. From the existing report, it has been well known that high dosage exposure to metabisulfite can cause serious damage to a large array of organs, including the lung [2,3], cardiovascular, and nervous systems [4,5]. Therefore, sodium metabisulfite residue in shrimp poses a serious threat to human health. With this regard, the usage of sodium metabisulfite in shrimp is still prohibited in China and many other countries in the world.

As for residual analysis in food, HPLC had been the “gold method” in most controlling laboratories [6,7,8]. Nevertheless, all these methods based on column chromatography were not able to provide a timely and non-destructive solution for the screening of sodium metabisulfite residue on shrimp. On the contrary, surface-enhanced Raman spectroscopy (SERS) is a simple and fast spectral analysis technology. Compared to other spectroscopic detection, SERS shows outstanding advantages in the following two aspects: (1) High sensitivity. The SERS effect enhances Raman scattering by molecules adsorbed on rough metal surfaces or by nanostructures such as noble metal (Ag or Au) nanoparticles, which is understood to be conditioned by the localization of detected molecules into abnormally strong nano-scale localized optical fields. Particularly, the enhancement factor can be as much as 10^10^ to 10^15^ if special geometric structures, the so-called “hot-spot” of nanoparticles, are formed. As reported by Nie, the optical responses of a single molecule and nanoparticles were recorded by SERS, showing intrinsic Raman enhancement factors on the order of 10^14^ to 10^15^ [9]. (2) Rich structural information. The featuring advantage of SERS is the sharp, fingerprint-like spectra pattern specific for the respective analyte. SERS spectra can give vibrational spectroscopic fingerprints from chemical and biological materials and therefore provide a comprehensive characterization tool to gain an understanding of the molecular structure [10,11]. Therefore, SERS offers high sensitivity and specificity in molecular identification and is a promising tool for the detection of adverse residues in food [12,13,14,15,16,17,18,19].

Though the unusual phenomenon of SERS was observed in 1977, there is still lots of controversy about its mechanism. Generally, two major theoretical explanations for the SERS mechanism have been proposed. The first is a chemical enhancement, which primarily involves the charge transfer mechanism. The other one is an electromagnetic enhancement that results from the amplification of the light intensity by the excitation of localized surface plasmon resonances. In both theories, the strength of SERS is critically dependent on the quality of the substrate material. A large array of SERS substrate materials had been proposed to increase the detectability of SERS as much as possible. Recently, emerging flexible SERS substrates as an alternative to colloidal and rigid SERS substrates have attracted remarkable attention [20,21,22]. More specifically, flexible SERS substrates highlight the advantage of easy sampling by wrapping or swabbing on irregular surfaces, which ideally facilitates the detection of chemical residues on food surfaces. In this way, analysis steps can be performed in a highly efficient way, and the interferences caused by co-extracted sample matrices can be perfectly circumvented. This opens a new horizon on the non-destructive and sensitive analysis of adverse residues on the surface of the food.

**Figure 1:**
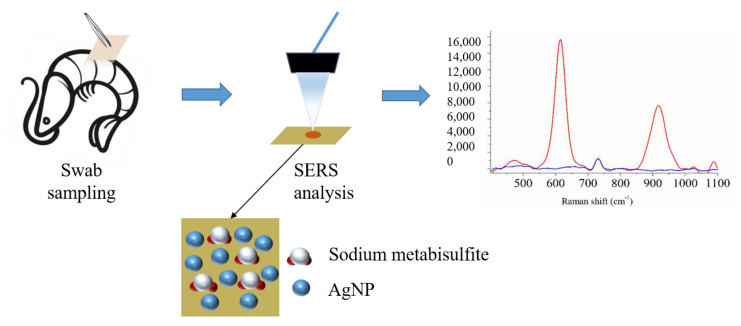
Schematic illustration of SERS detection of sodium metabisulfite residue on shrimp surface.

Compared to other flexible materials such as textiles, copy paper is the most commonly available flexible material ideally able to host nanoparticles of silver and gold, which is especially suitable for in-situ screening. Additionally, the copy paper itself does not generate any strong SERS signal, implying that background interference from the substrate material can be excluded. There have been many reports that evidenced that paper was able to facilitate on-site SERS detection of substance molecules in the field [23,24]. Loaded with Ag nanoparticle (AgNP), copy paper can be cut into any size and shape, bent, and folded [25]. Copy paper can also be covered on an irregular sample surface without destroying raw materials, which may reduce the number of “hot spots” during in situ detection [26]. Therefore, compared with other rigid substrate materials, copy paper-based flexible substrates displayed stronger detection ability and wider application on complex and irregular surfaces, allowing effective detection at low concentrations [27]. In this study, copy paper loaded with AgNP (AgNP−CP-) was fabricated in order to realize fast and simple screening of sodium metabisulfite on the surface of shrimp, which was illustrated in Figure 1.

## 2. Materials and Methods

### 2.1. Chemicals and Equipment

Silver nitrate (AgNO_3_, purity ≥ 99.8%), sodium citrate (C_6_H_5_Na_3_O_7_·2H_2_O, purity ≥ 99%), and sodium metabisulfite were purchased from Sinopharm Chemical Reagent Co., LTD (Beijing, China). A4 copy paper was purchased from Deli Ltd. (Shanghai, China). The magnetic heating stirrer-MS-H-ProA was from Dragon Laboratory Instruments Ltd. (Shanghai, China). The portable Raman spectra analyzer-ATR3110 workstation was from Optosky Photonics Ltd. (Xiamen, China). Ultra-pure water (conductivity: 1.08 μS/cm) was prepared by a Millipore Synergy system (Schwalbach, Germany). UV-vis spectrophotometer was from Jinghua Ltd. (Shanghai, China). Shrimp samples were purchased from a local supermarket.

### 2.2. Synthesis of AgNPs

The synthesis of AgNPs was principally based on the method proposed by Lee and Meisel [28] and characterized by UV-vis spectrophotometer and Scanning Electronic Microscopy. Briefly, 45.0 mg of AgNO_3_ was dissolved in 250 mL of ultra-pure water. The mixture was evenly stirred and then boiled. Afterward, 5 mL of 1% trisodium citrate solution was added drop by drop to the boiling mixture; meanwhile, the mixture was magnetically stirred during the whole process to ensure uniform heating. After boiling for 1 h, heating was stopped, and the solution was cooled down to room temperature with continuous stirring. A grey-green colloidal solution of AgNPs was obtained, which was refrigerated at 4 °C, sealed, and stored in the dark.

### 2.3. Fabrication of AgNP−CP

The blank copy paper was cut into 1 cm × 1 cm pieces, which were soaked in the as-prepared AgNPs colloid. After the AgNPs were evenly adsorbed on the paper substrates, they were taken out and put into a closed space to dry naturally and set aside for later use.

### 2.4. Preparation of Standard Solutions

Preparation of sodium metabisulfite standard solution: 0.05 g of sodium metabisulfite solid powder was dissolved in 5 mL of ultra-pure water to prepare a 10 mg/mL sodium metabisulfite standard solution. The standard solution was serially diluted to prepare 5, 3, 1, 0.2, and 0.1 mg/mL solutions, which were sufficiently shaken before use.

### 2.5. Preparation of Shrimp Samples

The SERS detection process of sodium metabisulfite in shrimp is shown in Figure 1. The different diluted solutions of sodium metabisulfite (prepared 5, 3, 1, 0.2, and 0.1 mg/mL) were sprayed on the shrimp surface and allowed to dry.

### 2.6. SERS Sampling and Measurement

As for SERS analysis of the standard solution of sodium metabisulfite, 10 μL of the solution was applied within an area of 1 cm^2^ square on clean glass. The liquid was allowed to dry at room temperature. Then, the AgNP-CP was wiped on the square area in order to sample the analyte onto the SERS substrate material. As for the analysis of the real shrimp sample, the AgNP-CP was wiped on the shrimp’s surface. After that, the quantitative measurement was performed with a portable Raman spectrometer, with an excitation wavelength of 785 nm and laser power at 80 mW. The acquisition time was 500 ms with one accumulation. The integration time is 8000 ms, the signal-to-noise ratio threshold is 3, and the intensity threshold is 1000. Spectral data recorded by the Raman spectrometer were processed using Origin 8.5 software. Each sample was measured three times to obtain the average value.

## 3. Results and Discussion

### 3.1. Fabrication and Characterization of AgNP-CP

**Figure 2:**
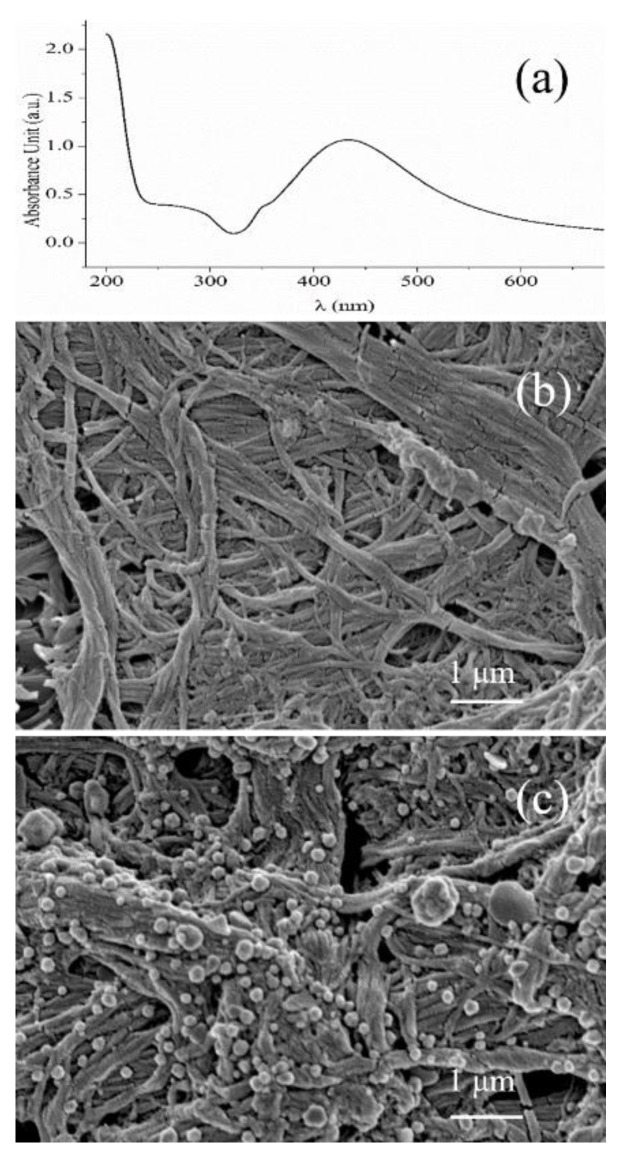
Ultraviolet absorption spectra of the AgNPs colloid (**a**); Microscopic structure of the blank copy paper (**b**) and copy paper loaded with AgNP by dipping (**c**).

In this study, the colloid of AgNPs was first prepared. The significant surface plasma effect of AgNPs can be characterized by their light absorption spectrum. As shown in Figure 2a, the light absorption spectrum of the raw AgNPs colloid after 20-fold diluting was continuous and displayed the maximum absorption peak at 440 nm, indicating that the AgNPs prepared in this work had the effectiveness and could be applied to the study of nanomaterials.

Then the flexible SERS substrate was obtained by simply dipping the copy paper into the colloid of AgNPs. After that, the color of the copy paper became gray, evidencing that the dispersed AgNPs were absorbed in the fiber structure of cellulose. The color change was resistant to rinsing, suggesting that absorption was strong enough. The electron microscope photographs enabled further insight into the morphology of the substrate. As shown in Figure 2b,c, lots of AgNPs uniformly anchored on the cellulose fiber structure can be observed after dipping, compared to that of the blank copy paper. This further evidenced the success of SERS substrate fabrication.

### 3.2. Usability Evaluation of the SERS Substrate

**Figure 3:**
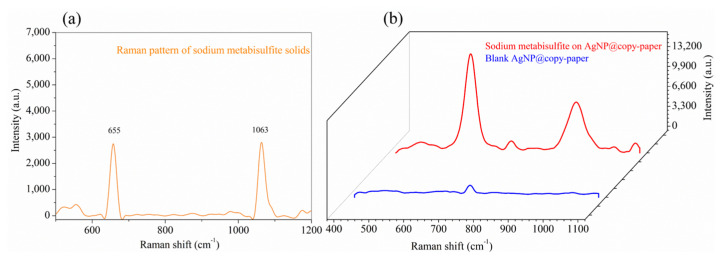
The Raman scattering spectrum of sodium metabisulfite (**a**); SERS spectra of the AgNP-CP with or without sodium metabisulfite standard solution (**b**).

In order to access the usability of the as-prepared substrate, SERS measurement to the standard solution of sodium metabisulfite was carried out. As shown in Figure 3, the blue line represents the blank substrate response, and the red line represents the SERS signal of the sodium metabisulfite standard solution (10 mg/mL). It was apparent that the blank copy paper was inactive to SERS since the spectrum is a flat line. On the other hand, the copy paper became highly SERS active after being loaded with AgNPs. The SERS signal of the sodium metabisulfite aqueous solution displayed two characteristic peaks at 927 and 620 cm^−1^, agreeing well with its Raman scatter pattern, comparatively shown in Figure 3a,b. The assignment of these characteristic peaks is listed in Table 1. Due to the electromagnetic coupling between S and Ag, S-O stretching vibration (symmetry + symmetry), and O-S-O symmetry, SO_2_ binding to the surface of AgNPs generated two strong and comparable characteristic peaks at 927 and 620 cm^−1^. Since the strength of the S-O tensile band can be variable, the characteristic peaks will deviate accordingly [29,30]. These results evidenced that SERS detection can provide an ambiguous tool for the identification and confirmation of metabisulfite ions on shrimp, even in the absence of the reference standard.

### 3.3. Analysis Sensitivity

**Figure 4:**
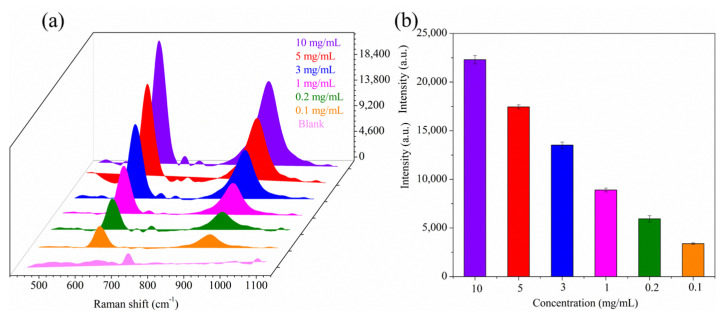
SERS spectra of AgNP-CP wipes of air-dried sodium metabisulfite solutions on glass plates (**a**); Error analysis of parallel detection for different concentrations of sodium metabisulfite standard solutions (0.1–10 mg/mL) (fingerprint peaks at 620 cm^−1^) (**b**).

In routine screening tasks, the primary concern is whether the shrimp were treated with sodium metabisulfite or not. Therefore, the sensitivity of detection is of crucial importance. In order to evaluate the detectability of the developed method, a glass plate was used as the blank control first. More specifically, sodium metabisulfite solutions of different concentrations were added dropwise to the glass plate and air-dried. Then, the spot on the glass plates was wiped with AgNP-CP for SERS measurement. As shown in Figure 4a, the characteristic SERS peaks at 620 and 927 cm^−1^ were clearly distinguished even at a low concentration of 0.1 mg/mL sodium metabisulfite. In addition, parallel analyses of different concentrations of sodium metabisulfite were performed to verify the reliability of the AgNP-CP detection system over a range of 0.1–10 mg/mL (Figure 4b). The results showed minor deviations in sensitivity with no obvious influence of the test solution concentration, evidencing good sensitivity and reliability of the analysis method. Moreover, the analysis sensitivity could be further enhanced by using other plasmonic Ag material with higher SERS activity [31,32,33].

### 3.4. Precision Evaluation of the Analysis

**Figure 5:**
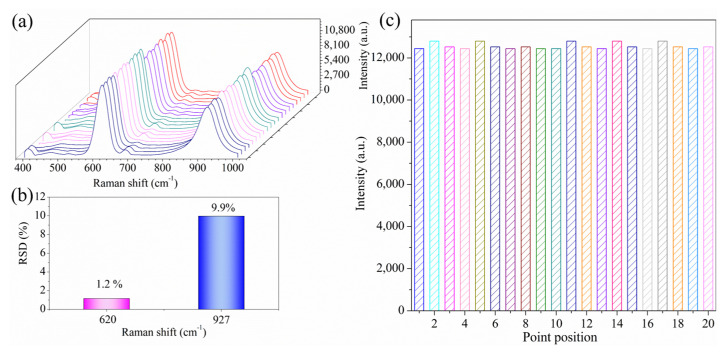
SERS spectrogram from 20 randomly selected points on a single piece of AgNP-CP (**a**), RSDS peak intensity at 20 random points, and (**c**) bar chart showing peak intensity data from the 20 random points (**b**).

The uniformity analysis of the detection was investigated using 1 mg/mL sodium metabisulfite solution as a probe and using the wiping method. A volume of 10 μL of the 1 mg/mL sodium metabisulfite solution was dropped onto the glass plate and wiped after drying. Then, 20 points were randomly selected on a single AgNP-CPAgNP-CP piece to collect the SERS spectra. As shown in Figure 5a, the intensity of fingerprint peaks at 620 and 927 cm^−1^ did not fluctuate significantly, and the SERS signal display rate reached 100% in 20 measurements, indicating the uniform distribution of the test substance on the AgNP-CP detection system. Figure 5b shows that there was almost no significant change in the intensity of the 620 cm^−1^ peak, and the relative standard deviation (RSD) was only 1.2%, indicating the good performance of the SERS detection system. Moreover, the data in Figure 5c shows that the intensity of the SERS response peak 620 cm^−1^ from 20 randomly selected points did not change significantly. These results showed that the AgNP-CP detection system is simple, reliable, and has good uniformity.

### 3.5. Stability Evaluation of the Analysis

**Figure 6:**
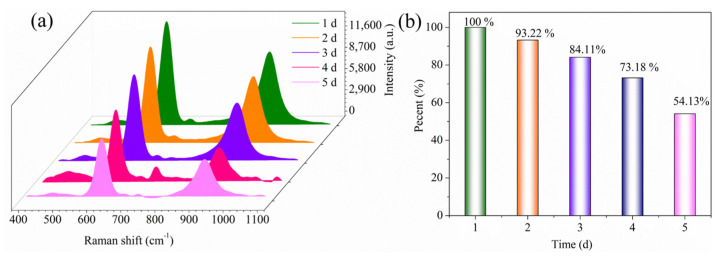
SERS spectrogram of 5-day stability test of AgNP-CP for sodium metabisulfite (**a**) and comparison of peak intensity at 620 cm^−1^ (**b**).

The stability test of the AgNP-CP was performed using the glass slide wiping test. AgNP-CP substrates of the same preparation batch were used for analysis. The soaked paper substrates were dried, placed in closed glassware, and then stored at 18 °C and 30% humidity in the dark. The interval of measurement was 24 h, and the test stability was checked for 5 days. For the wiping analysis method, we used 10 μL of 1 mg/mL sodium metabisulfite standard solution. The analysis of stability over five days is shown in Figure 6. The stability gradually decreased over time. On the fifth day, though the peak intensity (620 cm^−1^) reduced to about half of that on the first day, the characteristic peaks at 620 and 927 cm^−1^ were still clear and easy to distinguish. Such a descending trend might be attributed to the oxidation of silver over storage, suggesting that the SERS substrate was better to be used shortly after fabrication.

### 3.6. Analysis of Real Samples

**Figure 7:**
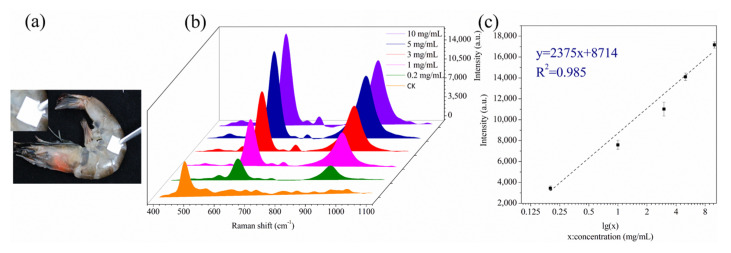
AgNP-CP wiping test on the shrimp surface (**a**), SERS sensitivity of the AgNP-CP wiping test, and (**c**) linear relationship between the intensity of 620 cm^−1^ peak and sodium metabisulfite concentration (**b**).

Figure 7a shows the SERS spectra of shrimp surfaces sprayed with sodium metabisulfite standard solutions of different concentrations. After allowing the sodium metabisulfite solution to dry naturally, the shrimp surface was sprayed with prepared ethanol aqueous solution, and the surface wiping test was performed with AgNP-CP, and SERS signals were recorded (Figure 7b). The intensity of the two characteristic peaks at 620 and 927 cm^−1^ became gradually weaker as the sodium metabisulfite concentration decreased. Due to the specific influence of the shrimp on the substrate, the detection of sodium metabisulfite solution only reached 0.2 mg/mL. Then, we used the SERS peaks for quantitative analysis and established a standard curve between the concentrations of the test samples and the intensity of the peak at 620 cm^−1^. The results are shown in Figure 7c. The standard curve was fitted to the 375x + 8714 (R^2^ = 0.985) equation. The LOD of the detection of sodium metabisulfite on the shrimp surface was 0.31 mg/kg. Compared with other SERS detection of sodium metabisulfite in recent years, our detection method is remarkably convenient, especially suitable for screening tasks (Table 2). A comparison of the data showed that the AgNP-CP has good sensitivity, and the test had a linear correlation of the standard curve for accurate and quantitative analysis. This suggested that the AgNP-CP assay can be used for the quantification of the residue of sodium metabisulfite on shrimp.

## 4. Conclusions

In this study, we developed a SERS method using a flexible AgNP-CP substrate material for the rapid determination of sodium metabisulfite on shrimp surface that can be conducted with a portable Raman spectrometer. AgNP-CP exhibited good adsorption uniformity, maintaining the reproducibility of the SERS results. The SERS detection sensitivity of AgNP-CP for sodium metabisulfite solution on the shrimp surface was 0.2 mg/mL, and the LOD value was 0.31 mg/kg. We also established a quantitative relationship between the intensity of the characteristic peak at 620 cm^−1^ and the sodium metabisulfite concentration. The linear fitting equation was 2375x + 8714 (R^2^ = 0.985), evidencing that this method could be used for quantitative screening. Generally, the proposed method demanded simple sample pre-treatment, short analysis time, and portable equipment, showing high simplicity and cost-effectiveness. Therefore, it might be suitable for on-site and non-destructive screening of the sodium metabisulfite residue on shrimp. However, the stability of the SERS activity of the substrate material over storage still needed to be further optimized.

## Figures and Tables

**Table 1:** Distribution of the two characteristic peaks of sodium metabisulfite.

Fingerprint Peak (cm^−1^)	Intensity	Signal Assignment
620	Strong	Symmetrical bending vibrations of O-S-O
927	Medium	Symmetrical and asymmetric S-O stretching vibrations

**Table 2:** Comparison of detection methods for sodium metabisulfite.

Method	Analyte	Sample Matrix	LOD	Reference
Near-infrared	Sodium metabisulfite	Fresh-cut potatoes	500 g/kg	[34]
Colorimetry	Sulfite	Foods	27.6 nM	[35]
RP-HPLC	Sodium metabisulfite	Drugs	95 mg/L	[36]
SERS	Sodium metabisulfite	Shrimp	0.31 mg/kg	This study

## Data Availability

The data will be available on request.

## References

[1] Robbins K.S., Shah R., MacMahon S., de Jager L.S. (2015). Development of a Liquid Chromatography–Tandem Mass Spectrometry Method for the Determination of Sulfite in Food. J. Agric. Food Chem..

[2] Wang X., Cao H., Guan X.L., Long L.H., Hu Z.L., Ni L., Wang F., Chen J.G., Wu P.F. (2016). Sulfite triggers sustained calcium overload in cultured cortical neurons via a redox-dependent mechanism. Toxicol. Lett..

[3] Han X., Zhu F., Chen L., Wu H., Wang T., Chen K. (2020). Mechanism analysis of toxicity of sodium sulfite to human hepatocytes L02. Mol. Cell. Biochem..

[4] Yao G.Y., Yun Y., Sang N. (2016). Differential Effects Between One Week and Four Weeks Exposure to Same Mass of SO_2_ on Synaptic Plasticity in Rat Hippocampus. Environ. Toxicol..

[5] Yao G., Yue H., Yun Y., Sang N. (2015). Chronic SO_2_ inhalation above environmental standard impairs neuronal behavior and represses glutamate receptor gene expression and memory-related kinase activation via neuroinflammation in rats. Environ. Res..

[6] Pizzoferrato L., Quattrucci E., Di Lullo G. (1990). Evaluation of an HPLC method for the determination of sulphiting agents in foods. Food Addit. Contam..

[7] Theisen S., Hansch R., Kothe L., Leist U., Galensa R. (2010). A fast and sensitive HPLC method for sulfite analysis in food based on a plant sulfite oxidase biosensor. Biosens. Bioelectron..

[8] Chung S.W.C., Chan B.T.-P., Chan A. (2008). Determination of free and reversibly-bound sulfite in selected foods by high-performance liquid chromatography with fluorometric detection. J. AOAC Int..

[9] Nie S., Emory S.R. (1997). Probing Single Molecules and Single Nanoparticles by Surface-Enhanced Raman Scattering. Science.

[10] Chen Y., Huang C., Jin Z., Xu X., Cai Y., Bai Y. (2020). HPTLC-bioautography/SERS screening nifedipine adulteration in food supplement based on Ginkgo biloba. Microchem. J..

[11] Chen Y., Huang C., Hellmann B., Xu X. (2020). HPTLC-Densitometry Determination of Riboflavin Fortified in Rice Noodle: Confirmed by SERS-Fingerprint. Food Anal. Methods.

[12] Pan C., Chen H.J., Lin Q., Luo S.H., Gu J.L., Ye S.Q., Zeng Y.M., Ren B., Tian Z.Q., Xue W.D. (2020). Evaluation of the SERS-based strategy in fast and on-site food safety inspection: Qualitative and quantitative analysis of trace unexpected herbicide in complicated herbicide matrix. J. Raman Spectrosc..

[13] Xu M.L., Gao Y., Han X.X., Zhao B. (2017). Detection of Pesticide Residues in Food Using Surface-Enhanced Raman Spectroscopy: A Review. J. Agric. Food Chem..

[14] Xin H., Namgung B., Lee L.P. (2018). Nanoplasmonic optical antennas for life sciences and medicine. Nat. Rev. Mater..

[15] Ma H., Liu S., Liu Y., Zhu J., Han X.X., Ozaki Y., Zhao B. (2021). In-situ fingerprinting phosphorylated proteins via surface-enhanced Raman spectroscopy: Single-site discrimination of Tau biomarkers in Alzheimer’s disease. Biosens. Bioelectron..

[16] Zhang Y., Yang Z., Zou Y., Farooq S., Li Y., Zhang H. (2023). Novel Ag-coated nanofibers prepared by electrospraying as a SERS platform for ultrasensitive and selective detection of nitrite in food. Food Chem..

[17] Bhaskar S., Srinivasan V., Ramamurthy S.S. (2023). Nd_2_O_3_-Ag Nanostructures for Plasmonic Biosensing, Antimicrobial, and Anticancer Applications. ACS Appl. Nano Mater..

[18] Xiong Y., Shepherd S., Tibbs J., Bacon A., Liu W., Akin L.D., Ayupova T., Bhaskar S., Cunningham B.T. (2023). Photonic Crystal Enhanced Fluorescence: A Review on Design Strategies and Applications. Micromachines.

[19] Beeram R., Vepa K.R., Soma V.R. (2023). Recent Trends in SERS-Based Plasmonic Sensors for Disease Diagnostics, Biomolecules Detection, and Machine Learning Techniques. Biosensors.

[20] Zhang D., Pu H., Huang L., Sun D.-W. (2021). Advances in flexible surface-enhanced Raman scattering (SERS) substrates for nondestructive food detection: Fundamentals and recent applications. Trends Food Sci. Technol..

[21] Wang Z., Zhang L., Chen Y. (2023). HPTLC+SRES screening of pesticide for point-of-care application as shown with thiram in juice. Food Chem. X.

[22] Wang S., Hao Q., Zhao Y., Chen Y. (2023). Two-Dimensional Printed AgNPs@Paper Swab for SERS Screening of Pesticide Residues on Apples and Pears. J. Agric. Food Chem..

[23] Pilot R. (2018). SERS detection of food contaminants by means of portable Raman instruments. J. Raman Spectrosc..

[24] Restaino S.M., White I.M. (2019). A critical review of flexible and porous SERS sensors for analytical chemistry at the point-of-sample. Anal. Chim. Acta.

[25] Xie J., Li L., Khan I.M., Wang Z., Ma X. (2020). Flexible paper-based SERS substrate strategy for rapid detection of methyl parathion on the surface of fruit. Spectrochim. Acta Part A Mol. Biomol. Spectrosc..

[26] Villa J.E.L., Quiñones N.R., Fantinatti-Garboggini F., Poppi R.J. (2019). Fast discrimination of bacteria using a filter paper–based SERS platform and PLS-DA with uncertainty estimation. Anal. Bioanal. Chem..

[27] Maddipatla D., Narakathu B.B., Atashbar M. (2020). Recent Progress in Manufacturing Techniques of Printed and Flexible Sensors: A Review. Biosensors.

[28] Lee P.C., Meisel D. (1982). Adsorption and surface-enhanced Raman of dyes on silver and gold sols. J. Phys. Chem..

[29] Chen M., Yang H., Rong L., Chen X. (2016). A gas-diffusion microfluidic paper-based analytical device (μPAD) coupled with portable surface-enhanced Raman scattering (SERS): Facile determination of sulphite in wines. Analyst.

[30] Deng Z., Chen X.X., Wang Y.R., Fang E.H., Zhang Z.G., Chen X. (2015). Headspace Thin-Film Microextraction Coupled with Surface-Enhanced Raman Scattering as a Facile Method for Reproducible and Specific Detection of Sulfur Dioxide in Wine. Anal. Chem..

[31] Wang Z.J., Li Q., Tan L.L., Liu C.G., Shang L. (2022). Metal-Organic Frameworks-Mediated Assembly of Gold Nanoclusters for Sensing Applications. J. Anal. Test..

[32] Verma A.K., Soni R.K. (2019). Silver nanodendrites for ultralow detection of thiram based on surface-enhanced Raman spectroscopy. Nanotechnology.

[33] Cheng D., He M., Ran J., Cai G., Wu J., Wang X. (2018). Depositing a flexible substrate of triangular silver nanoplates onto cotton fabrics for sensitive SERS detection. Sens. Actuators B Chem..

[34] Bai X., Xiao Q., Zhou L., Tang Y., He Y. (2020). Detection of Sulfite Dioxide Residue on the Surface of Fresh-Cut Potato Slices Using Near-Infrared Hyperspectral Imaging System and Portable Near-Infrared Spectrometer. Molecules.

[35] Xiang K., Chang S., Feng J., Li C., Ming W., Liu Z., Liu Y., Tian B., Zhang J. (2016). A colorimetric and ratiometric fluorescence probe for rapid detection of SO2 derivatives bisulfite and sulfite. Dye. Pigment..

[36] Ivković B., Brborić J., Dobričić V., Čudina O. (2019). Development and validation of a new isocratic RP-HPLC method for simultaneous determination of sodium metabisulfite and sodium benzoate in pharmaceutical formulation. Acta Chromatogr..

